# Dynamics changes of nutrients, flavor compounds, and beneficial microorganisms during Jianshui stinky tofu fermentation

**DOI:** 10.3389/fmicb.2026.1739952

**Published:** 2026-06-10

**Authors:** Jinming Meng, Zuxin Wu, Qingyu Ma, Hongshuo Zuo, Su Liu, Na Wu

**Affiliations:** Yunnan Province International Joint Laboratory of Green Food (China-Vietnam), School of Chemical and Resource Engineering, Honghe University, Mengzi, China

**Keywords:** fermentation, physicochemical parameters, soybean products, stinky tofu, volatile flavor

## Abstract

**Introduction:**

Traditional stinky tofu is predominantly produced via open fermentation, which is highly susceptible to environmental fluctuations. This leads to inconsistent product quality and severely restricts its industrial development and market promotion. This study aimed to systematically investigate the dynamic changes in basic physicochemical parameters, free amino acids, and volatile flavor compounds during the solid-state fermentation of Jianshui stinky tofu, to provide a scientific basis for its production quality control.

**Methods:**

We monitored the fermentation process of Jianshui stinky tofu and analyzed key quality indicators including titratable acidity, protein content, amino acid nitrogen, total polyphenols, and total flavonoids. Free amino acid profiles were determined using high-performance liquid chromatography (HPLC), and volatile flavor components were identified via headspace solid-phase microextraction coupled with gas chromatography-mass spectrometry (HS-SPME-GC-MS). Dominant microbial strains were isolated and identified through morphological observation and molecular biological methods.

**Results:**

During fermentation, titratable acidity increased to 0.80 g/100 g, while crude protein content decreased by 15.04%, and total amino acid nitrogen increased 4-fold due to continuous microbial metabolism. The bioactive components were significantly enhanced: total polyphenol and total flavonoid contents increased by 29.84% and 162.02%, respectively. Total free amino acids reached 402.07 mg/100 g, with essential amino acid content increasing 6-fold, indicating that fermented stinky tofu had significantly improved nutritional value and was more easily digestible. A total of 53 volatile flavor components were identified, with both the number and concentration of volatile compounds increasing markedly after fermentation. Ketones and heterocyclic compounds were the main contributors to the characteristic flavor of stinky tofu. Notably, phenol content was significantly higher than in other fermented soybean products, which was identified as the key substance forming its unique aroma. A high-protease-producing strain was isolated and identified as *Mucor circinelloides*.

**Discussion:**

This study systematically revealed the dynamic changes in quality and flavor formation during Jianshui stinky tofu fermentation. The findings provide a theoretical foundation for precise fermentation process control and the development of novel stinky tofu products.

## 1 Introduction

Soybean is one of the important food crops that has been under cultivation for 5,000 years and is widely grown worldwide, ranking fourth after corn, rice, and wheat in terms of cultivation area (Wang et al., 2022). Soybeans have a high nutrient content and a wide range of health benefits, including a protein content of approximately 40% of dry weight, a fat content of approximately 20%, and an abundance of vitamins and minerals (Vagadia et al., 2017). Soybean contains phytoactive components such as isoflavones, saponins, phytosterols, and phenolic acids, which are highly physiologically active and can lower blood cholesterol level, inhibit cancer cell growth, and enhance immunity (Cao et al., 2019; Rizzo, 2020). It also contains anti-nutritional factors such as phytic acid and trypsin inhibitors, lectins, and antigenic proteins, thus preventing the digestion, absorption, and utilization of nutrients by the body, and fermentation is the first choice to solve these problems (Tamang, 2015a; Prado et al., 2022).

Jianshui stinky tofu is one of the characteristic traditional foods in southwestern China and is popular among consumers for its nutrition, unique taste, and flavor, as well as its low price. The production process of Jianshui stinky tofu is complicated and involves 10 processes: screening, shelling, soaking, grinding, filtering, cooking, spotting, molding, blocking, and fermentation. After fermentation, soybean eliminates anti-nutritional inhibitory factors and decomposes insoluble macromolecular substances such as starch, protein, and fat into soluble low-molecular compounds polysaccharides, amino acids, fatty acids, and so forth. This dramatically improves the digestibility of its nutrients. More importantly, it retains the original functional substances such as soy isoflavones and oligosaccharides, significantly increases riboflavin and flavonoid glycosides, and new nutrients and bioactive substances after fermentation (Samtiya et al., 2020; Qiao et al., 2022). The production of stinky tofu relies on hydrolysis mediated by microorganisms and their associated enzymes. This hydrolysis process degrades soybean macromolecules and eliminates anti-nutritional factors (ANFs) in raw soybeans. It also generates abundant low-molecular-weight compounds, which greatly improves the quality and nutritional value of the final product. During the fermentation of stinky tofu, a series of complex biochemical reactions fully hydrolyze the nutrients in the raw materials and release a large number of esters, ketones, sulfur-containing compounds, and so forth. These metabolites ultimately form the characteristic unique flavor of stinky tofu (Diez-Simon et al., 2020). The unique and abundant flavor is one of the very important quality parameters of stinky tofu, which directly impacts the purchase demand of consumers.

The continuous development of the fermented soybean products market has led to many studies describing the role of the changes in nutritional composition and flavor substances in fermented soybean products. Compared with soybean, the antioxidant activity of fermented soybean products has dramatically improved, and the level of free amino acids, low-molecular-weight peptides, phenolic compounds, and other nutrients in the products is significantly enhanced (Xu et al., 2015; Gao et al., 2019). Tamang et al. found that traditional Indian fermented soy products were rich in nutritional value and low in fat and cholesterol contents. They were widely available in local recipes as cheap and easily absorbed plant proteins (Tamang, 2015b). The total phenol, isoflavone glycosides, and free amino acid contents of soymilk significantly increased after fermentation, and the antioxidant activity of the product was dramatically enhanced (Yamamoto et al., 2019). Shin et al. studied the Korean soybean paste and investigated whether microorganisms could convert proteins, fats, or carbohydrates in soybean into water-soluble substances and impart a rich flavor (Shin and Jeong, 2015). He et al. used solid-phase microextraction (SPME) to analyze the changes in volatile components in the brine of stinky tofu at different stages of the fermentation process. They showed that 3-methylindole was the main flavor substance throughout fermentation (He et al., 2016). Zhi et al. demonstrated that the types and contents of flavor substances in soybean paste increased significantly after fermentation (Tian et al., 2022). However, few studies involved a systematic research into the changes in nutrients and flavor substances during the fermentation process of Jianshui stinky tofu. In this study, we analyzed the changes in nutrients and flavor substances during the fermentation process of Jianshui stinky tofu. The findings might contribute to the field of the establishment of industry standards to promote standardized and large-scale production of stinky tofu. In turn, the study might promote the development of soybean farming and processing industries and contribute to the growth of the local economy.

## 2 Materials and methods

### 2.1 Chemicals and reagents

Rutin, gallic acid, and amino acid were of analytical standards and 2-octanol was chromatographically pure. All were purchased from the Sigma–Aldrich Company (MO, United States). PCA medium, RBC medium, PDA medium, and LB medium were purchased from Guangdong Huankai Microbial Technology Co. Ltd. (Guangzhou, China). Fungal Genomic DNA Extraction Kit was purchased from Fungal Genomic DNA Rapid Extraction Kit was purchased from Autobio Engineering Co. Ltd. (Shanghai, China). All other chemicals were of analytical grade and obtained from Sinopharm Chemical Reagent Co. Ltd. (Shanghai, China).

### 2.2 Sample collection

Bean curd blocks of uniform size (2 cm × 2 cm × 1 cm) and integrity were selected and placed in the traditional stinky tofu production workshop for natural fermentation. From the beginning of fermentation day 0 to the end of fermentation day 6, an appropriate amount of stinky tofu was collected daily for various physicochemical analyses. All samples underwent triplicate testing for the physicochemical analysis.

### 2.3 Physicochemical property analysis

#### 2.3.1 Analysis of titratable acidity, pH, and moisture, ash, crude protein, and amino acid nitrogen contents

The moisture and ash content of stinky tofu were determined by the direct drying and burning methods. Stinky tofu (5 g) was weighed in 50 mL of distilled water and homogenized for 30 s, and its pH was measured. The homogenate was filtered through a filter paper and titrated with 0.1 M of standard NaOH solution with 0.1 phenolphthalein solution as an indicator. The homogenate was filtered, and the amino acid nitrogen content of the samples was determined by the formaldehyde titration method. The crude protein content of the samples was determined by the Kjeldahl nitrogen determination method (Yang et al., 2021a).

#### 2.3.2 Analysis of the total phenol content

The Folin-phenol colorimetric method with few modifications was used to determine the total phenolic content (Xu et al., 2015). Lyophilized sample powder (0.2 g) was weighed, mixed with 5 mL of 70% ethanol solution, sonicated for 30 min at 70 °C, cooled to 20 °C, and centrifuged to collect the clear solution. The residue was repeatedly extracted with 5 mL of 70% ethanol solution, and the supernatant was combined and fixed to 10 mL. Then, 0.5 mL of the diluted extract was shaken well with 2.5 mL of forskolin reagent and left for 5 min, and then 2.0 mL of a 7.5% Na_2_CO_3_ solution was added and shaken well. The reaction was carried out at room temperature and protected from light for 60 min, and the absorbance value was measured at 765 nm. The total phenolic content of the sample was calculated according to the standard curve of gallic acid, and the results were expressed as gallic acid equivalent (mg/g).

#### 2.3.3 Analysis of the total flavonoid content

The NaNO_2_-Al (NO_3_)_3_ color development method with slight modifications was used to determine the total flavonoid content (Guo et al., 2023). Then, 1.0 g of lyophilized sample powder was accurately weighed and placed in a 50-mL conical flask, and 25 mL of a 75% ethanol solution was added. The sample was extracted by ultrasonic extraction for 2 h and filtered, and the filtrate was collected. Subsequently, 2.0 mL of the diluted sample extract was aspirated and placed in a 10-mL volumetric flask. First, 0.30 mL of 5% NaNO_2_ solution was added, shaken well, and left for 6 min. Then, 0.30 mL of 10% Al (NO_3_)_3_ solution was added, shaken well, and left for 6 min. Further, 4.00 mL of 4% NaOH solution was added, and then 30% ethanol solution was added to fix the volume to the scale, shaken well and left for 6 min. The absorbance was measured at a wavelength of 510 nm for 15 min. The total flavonoid content of the sample was calculated according to the standard curve of rutin, and the results were expressed as rutin equivalent (mg/g).

#### 2.3.4 Analysis of free amino acid content

The free amino acid content of stinky tofu was determined using an amino acid autoanalyzer (Mann-Moble, Berlin, Germany). A 0.1 g of the sample was weighed into a 10-mL centrifuge tube, 5 mL of a 4% sulfosalicylic acid solution was added and mixed by vortexing, the mixture was centrifuged at 10,000 rpm for 10 min, and the supernatant was filtered through a 0.22-μm filter before determination (Chen et al., 2023).

### 2.4 Analysis of volatile components

#### 2.4.1 Extraction of volatile components

The volatile components of the stinky tofu samples on different fermentation days were collected using headspace SPME. A 3-g sample was weighed into an SPME vial, 7 mL of distilled water and 0.5 g of NaCl were added, and 10 L of a 0.82 mg/mL 2-octanol solution was added as an internal standard. The sample was equilibrated in water at 40 °C for 15 min, and a 50/30 m divinylbenzene/carbene/polydimethylsiloxane SPME fiber extraction head was inserted into the vial and adsorbed for 40 min before injection into a gas chromatograph–mass spectrometer (GC–MS) for detection and analysis.

#### 2.4.2 GC–MS analysis

The extracted flavors were analyzed using a GC–MS (Shimadzu, Tokyo, Japan) equipped with a DB-1701 column (30 m × 0.25 mm × 0.25 μm). The chromatographic conditions were as follows: the inlet temperature was 240 °C, the carrier gas was helium, and the column flow rate was 1.0 mL/min. The initial temperature of the column chamber was 40 °C, which was maintained for 2 min, then increased to 150 °C at a rate of 5 °C/min, maintained for 3 min, then increased to 240 °C at a rate of 10 °C/min, and maintained for 18 min. The mass spectrometry conditions were as follows: The ionization mode was an electron ionization source, the electron energy was 70 eV, and the mass scan range was 30–500 m/z. The names of the volatiles were determined by comparison with the National Institute of Standards and Technology (NIST 14) mass spectrometry library. The content of each volatile compound was calculated using the internal standard method, with the formula as follows:


$$C_{i}=\frac{A_{i}\times C_{\text{is}}\times V_{\text{is}}}{A_{\text{is}}\times m}$$

Where:

*C_i_*: Content of the target volatile compound (μg/kg).

*A_i_*: Peak area of the target volatile compound.

*C_is_*: Concentration of the internal standard (μg/mL).

*V_is_*: Volume of the internal standard added to the sample (mL).

*A_is_*: Peak area of the internal standard.

*m*: Mass of the stinky tofu sample (kg).

### 2.5 Microbiological analysis

#### 2.5.1 Analysis of microbial colony counts

Accurately weigh 25 g of Jianshui stinky tofu samples from different fermentation stages, transfer into a conical flask, add 225 mL of sterile normal saline, and homogenize for 1–2 min to prepare a 10^−1^ stock dilution. Serial decimal dilutions were subsequently prepared with sterile normal saline, and the final appropriate dilutions (10^−1^ to 10^−5^) were selected for microbial enumeration. For each dilution, 0.1 mL of the diluted sample was spread in triplicate onto Plate Count Agar (PCA), Rose Bengal Chloramphenicol Agar (RBC), and Potato Dextrose Agar (PDA) plates, respectively. PCA plates were incubated at 36 °C for 48 h, while RBC and PDA plates were incubated at 28 °C for 72 h. Plates with colony counts within the optimal valid range were selected for final enumeration.

#### 2.5.2 Screening of protease-secreting microorganisms

The isolated and purified dominant bacteria, yeast, and fungi were separately inoculated into LB, YPD, and PDA liquid medium. The inoculated LB liquid medium was incubated at 36 °C and 200 r/min for 24 h, while the inoculated YPD and PDA liquid medium were incubated at 28 °C and 200 r/min for 48 h to obtain the corresponding seed culture. 0.1 mL of seed culture was, respectively, aspirated by the perforation method and inoculated into the protease screening medium. The medium was incubated at 36 °C for 24–72 h, and the diameters of the colonies and the clear zones were measured.

The obtained strains with clear zones were subjected to the above operation to prepare the seed cultures. Inoculate 1 mL of seed culture into 50 mL fermentation medium and ferment at 36 °C and 200 r/min for 72 h. After the fermentation was completed, centrifuge the fermentation liquid at 10,000 r/min at 4 °C for 10 min and take the supernatant. Take 100 μL of the supernatant and use a neutral protease assay kit to detect its enzyme activity. One unit of protease activity (U/g) was defined as the amount of enzyme that catalyzed the release of 1 μmol of tyrosine from casein per minute per gram of sample under the specified assay conditions.

#### 2.5.3 Identification of fungal strains

The DNA of mold M1-3 was extracted, isolated and purified using the fungal genomic DNA extraction kit. The 16S rDNA of the strain was performed by PCR using the universal fungal primers ITS1 and ITS4. The PCR products were analyzed via 1% agarose gel electrophoresis and subsequently then sent to Bioengineering (Shanghai) Co., Ltd. for sequencing. The sequencing results were submitted to the NCBI database for BLAST alignment to identify the most closely related sequences for each strain. A phylogenetic tree was constructed using MEGA7.0 software.

### 2.6 Statistical analysis

All the analyzed indicators of the samples were obtained by replicating triple tests, and the test results were expressed as mean ± standard deviation. Origin 2017 software (OriginLab, MA, United States) was used to process the experimental data and plot images. Significance and principal component analyses were performed using the SPSS Statistics 19.0 software (IBM, NY, United States), and differences were considered significant at *p* < 0.05.

## 3 Results and discussion

### 3.1 Physicochemical properties of stinky tofu during fermentation

**Figure 1:**
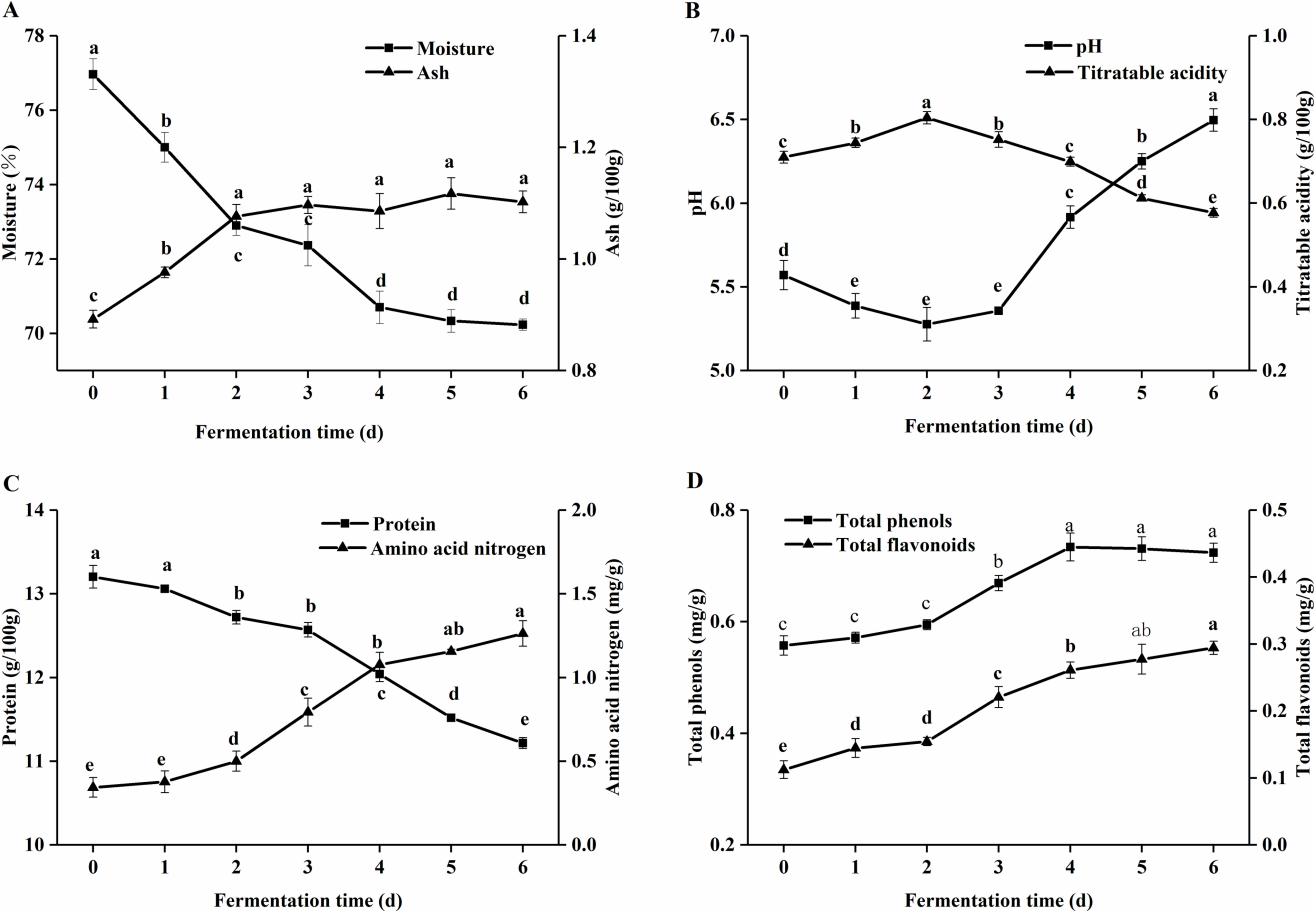
Changes in physicochemical parameters during the fermentation of stinky tofu. Moisture and ash **(A)**, pH and titratable acidity **(B)**, protein and amino acid nitrogen contents **(C)**, and total phenol and total flavonoid contents **(D)**. Different letters indicate significant differences (*p* < 0.05).

The moisture content of stinky tofu showed a decreasing trend during fermentation (Figure 1A). The high moisture content of tofu and the lower ambient humidity exacerbated the diffusion of water from tofu into the air in the initial stage of fermentation (Wu et al., 2018), which resulted in a rapid decrease (*p* < 0.05) in the water content of stinky tofu. During the first 3 days of fermentation, the water content of stinky tofu decreased from 77.69 to 70.70%. From the fourth day of fermentation, the moisture content decreased slowly (*p* > 0.05). The changes in the tofu were observed and recorded. A microbial mucous film formed on the surface of stinky tofu from the third day of fermentation. This film retarded the diffusion rate of water from stinky tofu to the ambient air. Moreover, the reduction in the water content caused the hardening of the surface of stinky tofu, further preventing water loss in the late fermentation stage (Suleman et al., 2020). The moisture content was maintained at about 70%. On the contrary, the variation in the ash content gradually increased in the early stage and stabilized in the later stage (Yao et al., 2021). These results were due to the decrease in moisture and the proliferation of microorganisms. The pH of stinky tofu decreased from 5.57 to 5.28 (range 1–3 days), and the titratable acid content rose to 0.80 g/100 g in the early stage of fermentation (Figure 1B). This was attributed to the catabolism of proteins, fats, and carbohydrates in tofu into free amino acids and organic acids with the growth of microorganisms (Ji, 2020; Yao et al., 2021). In turn, the pH of the stinky tofu increased significantly, and the titratable acid content decreased continuously after 3 days of fermentation. A possible explanation for this might be the oxidative decomposition of some amino acids into nitrogenous substances, such as ammonia and urea, by microorganisms. Meanwhile, urea was further hydrolyzed to ammonia, and the acids in stinky tofu were neutralized by ammonia, causing an increase in pH (Zhang et al., 2014). With the decrease of fermentable sugars in stinky tofu, some microorganisms utilized organic acids as a carbon source to sustain their metabolism, which resulted in a decrease of titratable acid content (Vermeulen et al., 2012).

Amino acid nitrogen was composed of short peptides and amino acids that originated from the enzymatic digestion of proteins and was commonly used as an indicator of the degree of fermentation (Zhao et al., 2021). The protein content decreased while the amino acid nitrogen content increased throughout the fermentation process (Figure 1C). The results of this experiment were consistent with the fermentation process of sufu (Yao et al., 2021). The decrease in the protein content was more obvious in the middle and late stages of fermentation. This result had several possible explanations. The reduction in water content led to an increase in salt concentration within the stinky tofu matrix. This elevated salt concentration disrupted the protein structure of stinky tofu, rendering it more susceptible to degradation by proteases. Another reason was that the proliferation of microorganisms led to an increase in protease secretion, which promoted protein degradation (Liu et al., 2021). From this data, it could be concluded that the degradation of proteins was continuous throughout the fermentation process.

### 3.2 Variations in total phenolic and total flavonoid contents of stinky tofu during fermentation

The graph showed a slight increase (*p* > 0.05) in the total phenolic content at the beginning of fermentation. The findings of the present study were consistent with those of Smith and Dulf who examined the effect of *Aspergillus niger* on the phenolic contents in *Sambucus* berry pomaces (Dulf et al., 2015). A probable explanation was that the presence of polyphenols in cells, mainly in the membrane-bound form, and the low number of microorganisms combined to result in the low concentration of polyphenols (Dulf et al., 2016). On the fourth day of fermentation, the total phenolic content peaked at 0.73 mg/g (Figure 1D). These results were consistent with those of other studies and suggested that fermentation could significantly increase the total phenolic content (Xiao et al., 2015). This was attributed to microorganisms secrete phenylalanine ammonia-lyase (PAL) and tyrosine ammonia-lyase (TAL), during fermentation. These enzymes catalyzed the deamination of L-phenylalanine and L-tyrosine. The reaction produced cinnamic acid and p-coumaric acid. These intermediate products underwent further modification via hydroxylation and methylation reactions. They were converted into a series of phenolic acids, such as caffeic acid and ferulic acid. These end products represented key contributors to the total phenolics (Wang et al., 2018; Fan et al., 2025). The slow decline (*p* > 0.05) in the total phenolic content at the later stages of fermentation might be ascribed to the secretion of relevant enzyme systems by microorganisms to hydrolyze the released phenolic substances into small molecules for microbial use because of the decrease in sugars content (Li et al., 2021; Guo et al., 2023).

Published studies and scientific reviews established that flavonoids had a strong antioxidant capacity, mainly via scavenging superoxide radicals, and soy products were an excellent source of flavonoid ingestion (Zaheer and Humayoun Akhtar, 2017). The total flavonoid content of stinky tofu increased consistently throughout the fermentation process, and it was 2.6 times higher than that at the end of fermentation (Figure 1D). Similarly, Lu xu et al. also reported an exponential increase in the content of total flavonoids in the beneficial health components of fermented soy products marketed in China (Xu et al., 2015). The main flavonoid components in the tofu matrix were bound glycosides (such as daidzin and genistin). These bound glycosides showed low detection response in conventional component assays. During fermentation, microorganisms secreted β-glucosidase, which specifically hydrolyzed the O-glycosidic bonds of these flavonoid glycosides. It released flavonoid aglycones, including daidzein and genistein. These aglycones exhibited higher detection efficiency and biological activity. This biochemical conversion drove the sharp increase in total flavonoid content (Di Gioia et al., 2014; Chen et al., 2020).

### 3.3 Changes in free amino acids in stinky tofu during fermentation

**Figure 2:**
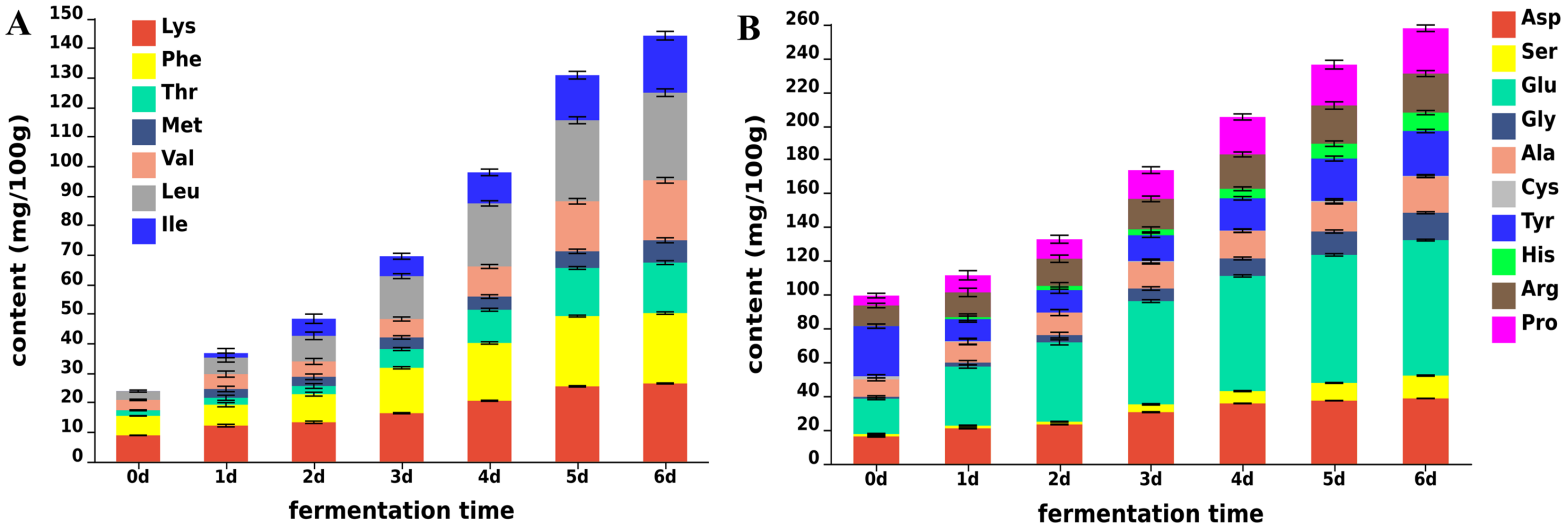
Variations in free amino acid content in stinky tofu at different fermentation times. Essential amino acids **(A)**, non-essential amino acid **(B)**.

The proteins in tofu were progressively hydrolyzed into amino acids by intracellular and extracellular enzymes during the fermentation process, and these free amino acids played a crucial role in the nutrition, taste, and flavor of stinky tofu (Luo et al., 2017). During the fermentation of stinky tofu, 17 common amino acids were detected. Except for cysteine, the content of all amino acids increased with extended fermentation time (Figure 2). This finding was in agreement with that of Wu et al. (2020). In this experiment, the total amino acid content in stinky tofu increased from 109.30 mg/100 g of tofu to 402.07 mg/100 g at the end of fermentation, which was a sixfold increase. The total essential amino acid content in stinky tofu increased from 23.90 mg/100 g of tofu to 144.29 mg/100 g at the end of fermentation, which was an increase of six times. This indicated that the amino acid content was significantly increased (*p* < 0.05), and the nutritional value of fermented tofu was greatly enhanced. During the fermentation process of stinky tofu, the elevated glutamic acid content reacted with salt to form monosodium glutamate (MSG). This flavor-active compound markedly improved the umami freshness of stinky tofu, making a substantial contribution to its overall taste profile (Zhang et al., 2021). Asp also enhanced the fresh and salty taste of stinky tofu and masked its bitterness (Chen et al., 2023). These two amino acids accounted for 29.56% of the total free amino acid content at the end of fermentation and were the major amino acids. The content of sweet amino acids (Thr, Ser, Gly, Ala, Lys, Pro) increased four times compared with that of tofu at the end of fermentation, accounting for 30.25% of the total free amino acid content. The accumulation of amino acids not only improved the taste of stinky tofu but also provided the material basis for the formation of aroma substances in the following fermentation process (Sun et al., 2013).

### 3.4 Changes in volatile flavors in stinky tofu during fermentation

**Figure 3:**
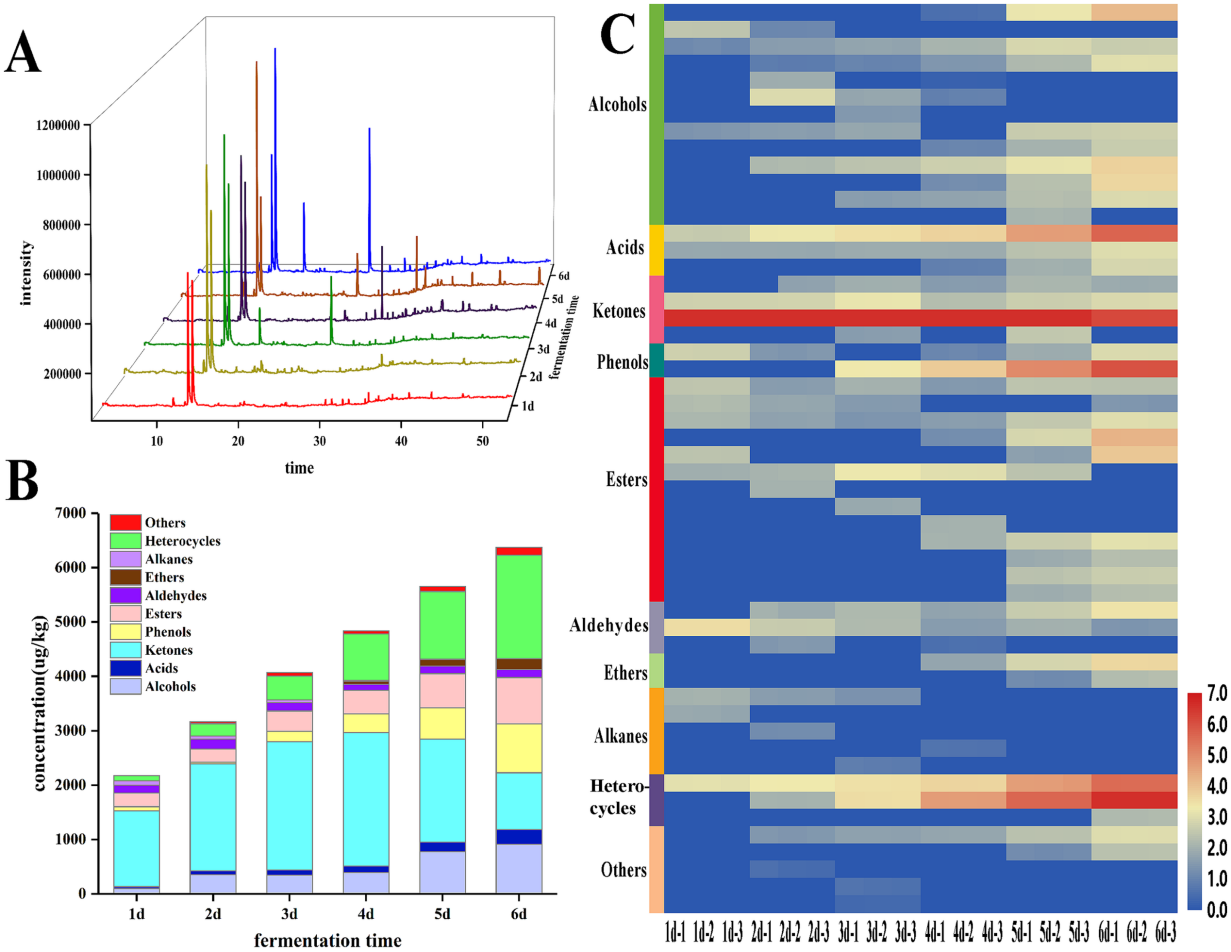
Analysis of volatile flavor compounds during the fermentation of stinky tofu. The total ion chromatogram **(A)**; histogram of flavor substance concentration **(B)**; heat map of the volatile compounds **(C)**.

The results revealed that approximately 53 volatile flavor components were identified, including 13 alcohols, 3 acids, 4 ketones, 2 phenols, 13 esters, 3 aldehydes, 2 ethers, 5 alkanes, 3 heterocycles, and 5 other compounds during the fermentation of stinky tofu (Figure 3A). The main flavor substances in stinky tofu in the pre-fermentation stage were 3-octanone and 2-octanone. The contents of both significantly decreased, while the contents of acetic acid, phenol, 2-pentylfuran, and indole significantly increased with the extension of fermentation time. The flavor substances of stinky tofu were mainly ethanol, acetic acid, 2-octanone, phenol, 2-pentylfuran, and indole at the end of fermentation. The total content of volatile flavor compounds in stinky tofu increased nearly threefold, from 2244.75 μg/kg on the first day of fermentation to 6704.45 μg/kg at the end of fermentation (Table 1). Figure 3C is a heat map of the change in flavor substance content during the fermentation of stinky tofu. The results showed that the blue area was occupied mainly in the first 3 days of fermentation, indicating that the types and content of flavor substances were lower. From the fourth day of fermentation, the blue areas decreased and the yellow and orange-red areas increased significantly. At the end of fermentation, the yellow and red areas accounted for the majority, showing a significant increase in the content and types of flavor compounds in stinky tofu. It seemed possible that these results were due to microbial reproduction, colony succession, and metabolism (Gao et al., 2020).

**Table 1:** Changes in the content and composition of volatile flavors in stinky tofu in different fermentation stages.

Components	Content of different fermentation times (μg/kg)					
	1 day	2 days	3 days	4 days	5 days	6 days
Alcohols						
Ethanol	–	–	–	10.88 ± 0.56c	140.91 ± 0.44b	216.43 ± 1.28a
1–Octanol	60.65 ± 0.79a	20.74 ± 0.34b	–	–	–	–
Eolinol	15.76 ± 1.00f	36.6 ± 0.48e	49.13 ± 0.95d	76.55 ± 0.46b	107.08 ± 0.25a	67.46 ± 0.67c
Terpinen–4–ol	–	14.27 ± 0.40e	21.33 ± 0.40d	39.01 ± 0.24c	60.95 ± 0.35b	94.22 ± 0.46a
2–Borneol	–	51.96 ± 0.01a	–	3.84 ± 0.16b	–	–
Terpenol	–	126.57 ± 0.02a	51.43 ± 0.94b	21.62 ± 1.09c	–	–
1–Heptanol	–	–	37.08 ± 0.42a	–	–	–
2–Nonanol	21.83 ± 1.11e	36.82 ± 0.69d	51.54 ± 0.60c	–	87.72 ± 0.44a	72.53 ± 0.79b
n–Hexanol	–	–	–	24.52 ± 0.64c	52.64 ± 0.02b	66.78 ± 0.01a
1–Nonanol	–	66.83 ± 1.07e	92.99 ± 0.81d	129.3 ± 0.40c	134.83 ± 0.70b	158.7 ± 0.90a
3–Octanol	–	–	–	30.91 ± 0.82c	69.54 ± 0.03b	149.27 ± 0.04a
1–Octen–3–ol	–	–	41.6 ± 0.71d	53.78 ± 0.33c	66.97 ± 1.38b	86.57 ± 0.51a
Chlorophyl alcohol	–	–	–	–	54.96 ± 0.88a	–
Acids						
Acetic acid	67.23 ± 1.25f	158.15 ± 0.81e	218.79 ± 1.32d	286.8 ± 1.79c	409.3 ± 0.79b	646.13 ± 1.11a
2–Methylbutyric acid	33.58 ± 0.01f	46.75 ± 0.6e	58.07 ± 0.67d	64.51 ± 0.60c	72.82 ± 0.78b	91.12 ± 0.66a
Propionic acid	–	–	–	20.71 ± 0.60c	49.59 ± 0.50b	80.1 ± 0.77a
Ketones						
4–(2,2,6–trimethyl–7–oxabicyclo[4.1.0]hept–1–yl)–3–buten–2–one	–	40.82 ± 0.45d	61.84 ± 0.46a	44.28 ± 0.49c	57.28 ± 0.53b	34.56 ± 0.57e
3–Octanone	76.75 ± 0.87f	109.32 ± 0.99b	172.72 ± 0.99a	93.46 ± 0.66c	91.29 ± 0.60d	85.03 ± 0.59e
2–Octanone	1319.19 ± 5.34e	1832.9 ± 2.24c	2084.41 ± 3.32b	2315.4 ± 2.60a	1663.88 ± 3.50d	922.93 ± 2.63f
2–Nononone	–	–	41.7 ± 0.93b	–	80.63 ± 0.60a	–
Phenols						
2, 4–Di–tert–butylphenol	74.85 ± 0.98b	31.55 ± 1.16d	–	25.54 ± 1.22e	46.44 ± 0.68c	85.97 ± 0.75a
Phenol	–	–	192.78 ± 0.76d	316.8 ± 0.55c	531.12 ± 0.98b	816.49 ± 0.73a
Esters						
Dinonyl sebacate	61.12 ± 0.38c	36.13 ± 0.62f	65.53 ± 0.63b	41.33 ± 0.49e	74.88 ± 0.86a	53.81 ± 0.55d
Diisobutyl phthalate	50.41 ± 1.24a	45.25 ± 0.56b	38.8 ± 0.22c	–	–	21.21 ± 0.87d
Ethyl palmitate	46 ± 0.37c	41.2 ± 0.75d	33.79 ± 0.53e	46.96 ± 0.17c	80.98 ± 0.49b	91.41 ± 1.25a
Ethyl oleate	–	–	–	31.14 ± 0.34c	107.56 ± 1.06b	234.47 ± 1.23a
Ethyl linoleate	58.47 ± 0.01b	–	–	–	34.89 ± 0.82c	182.6 ± 0.63a
Octyl p–methoxycinnamate	39.12 ± 0.43e	61.38 ± 1.01d	180.73 ± 0.27a	169.38 ± 0.98b	73.05 ± 0.43c	–
Bornyl acetate	–	58.29 ± 0.29a	–	–	–	–
Ethyl heptadecanoate	–	–	54.6 ± 0.57a	–	–	–
Dibutyl phthalate	–	–	–	73.24 ± 0.69a	–	–
N–nonyl chloroformate	–	–	–	74.31 ± 0.58c	88.69 ± 0.57b	96.58 ± 1.42a
Dibutyl phthalate	–	–	–	–	45.91 ± 1.84b	51 ± 0.82a
Butyl phthalyl glycerol	–	–	–	–	73.2 ± 1.90a	68.02 ± 0.52b
Dibutoxy–ethyl adipate	–	–	–	–	47.74 ± 1.10b	51.32 ± 0.97a
Aldehydes						
Nonaldehyde	–	53.04 ± 4.47d	76.21 ± 0.28c	54.14 ± 0.63d	86.99 ± 0.80b	125.82 ± 0.33a
Decal	143.27 ± 0.79a	94.83 ± 0.87b	78.29 ± 0.70c	45.07 ± 0.57d	51.12 ± 0.42c	20.86 ± 0.60e
Dodecal	–	32.34 ± 1.19a	–	10.95 ± 0.58b	–	–
Ethers						
Dimethyl trisulfide	–	–	–	56.13 ± 0.01c	104.38 ± 0.66b	148.15 ± 0.53a
Dimethyl tetrathioether	–	–	–	–	20.71 ± 0.56b	50.13 ± 1.52a
Alkanes						
Cycloheptane	41.72 ± 0.73a	36.07 ± 0.59b	32.06 ± 0.02c	–	–	–
Phytane	33.4 ± 1.58a	–	–	–	–	–
Cetane chloride	–	24.03 ± 0.31a	–	–	–	–
Heptamethylnonane	–	–	–	12.65 ± 0.41a	–	–
Tetracosane	–	–	16.56 ± 1.00a	–	–	–
Heterocycles						
2–Pentylfuran	101.4 ± 1.01f	166.44 ± 1.32e	215.21 ± 0.65d	278.85 ± 0.77c	421.24 ± 17.81b	589.6 ± 2.17a
Indole	–	59.16 ± 0.56e	223.76 ± 1.92d	579.78 ± 1.76c	827.9 ± 2.36b	1268.82 ± 1.14a
N–(2–Aminoethyl)morpholine	–	–	–	–	–	47.43 ± 0.79a
Others						
Octodrine	–	31.42 ± 0.87e	45.41 ± 0.63d	56.56 ± 0.75c	71.9 ± 0.23b	90.83 ± 0.78a
beta–Elemene	–	–	–	–	21.17 ± 0.71b	54.57 ± 0.50a
Caryophyllene	–	7.54 ± 1.05a	–	–	–	–
2–Cyclohexylethylamine	–	–	10.88 ± 0.85a	–	–	–
Decene	–	–	6.92 ± 0.18a	–	–	–
Total content	2244.75 ± 3.73f	3306.76 ± 20.82e	4254.17 ± 2.96d	5077.49 ± 4.16c	5869.35 ± 20.34b	6704.45 ± 2.61a

Means with different letters (a–f) in the same row are significantly different (*p* < 0.05).

### 3.5 Analysis of main flavor substances

Ketones were the main flavor substances in the fermentation process of stinky tofu, and their contents first increased and then decreased as the fermentation proceeded, with their percentage decreasing from 64.10 to 16.35% (Figure 3B). Four kinds of ketones were detected as 4–(2,2,6–trimethyl–7–oxabicyclohept–1–yl) – 3–buten–2–one, 3–octanone, 2–octanone, and 2–nonone, of which 2–octanone accounted for the highest proportion, followed by 3–octanone (Table 1). 2–Octanone exhibited an apple aroma, while 3–octanone exhibited a strong mushroom aroma. The enzymatic reactions of fungi to lipids or amino acids, or the Maillard reaction, could produce large amounts of ketones in the fermentation process of stinky tofu (Liu et al., 2018). Although the threshold of most aliphatic ketones was higher, their abundance conferred a unique flavor to Jianshui stinky tofu.

As shown in Figure 3B, the concentration of heterocycles markedly increased during the fermentation of stinky tofu and was the major flavor substance of stinky tofu at the end of fermentation, mainly composed of 2–pentylfuran and indole. 2–Pentylfuran displayed soy, fruity, earthy, green, and vegetable–like aromas and was also the main flavor substance in natto (Liu et al., 2018). Furan was generated by nonenzymatic chemical reactions of various precursors such as acetylacetone, acetylacetone, free amino acids, and ammonia (Zheng et al., 2022). Yang et al. similarly identified it as the main flavor substance in fermented soybean whey tofu (Yang et al., 2021b). Indole had a strong unpleasant odor, an animal smell, and an orange and jasmine aroma when diluted, with a very low aroma threshold. Bacteria reversibly catalyzed the conversion of tryptophan into indole, pyruvate, and ammonia by secreting tryptophanase. Lee et al. also detected the presence of indole in stinky tofu (Lee and Lee, 2010).

Alcohols accounting for one–sixth of all volatile flavor compounds were among the most important volatile flavor compounds, mainly ethanol, 1–nonanol, and 3–octanol, at the end of fermentation (Table 1). Alcohols are known to be the by-products of lipid oxidation, mainly from the oxidative degradation of polyunsaturated fatty acids (Zhu et al., 2019), leading to the emission of an unpleasant odor (Yao et al., 2022). 1–Nonanol had a fatty wax aroma with rose wax and fruitiness (Jia et al., 2019). 3–Octanol was formed from the hydroperoxide isomer of linoleic acid by the hydroperoxide cleavage enzyme and had a strong mushroom–like odor and grassy aroma (Yu et al., 2022).

The content of phenolic substances showed a remarkably large increase, accounting for 14.15% of the total volatile flavor compounds. Phenols are primarily obtained from the microbial decomposition of tyrosine (Cui et al., 2022). The data presented an interesting observation that the phenol content in other fermented soybean products was generally low; however, in Jianshui stinky tofu, it was extremely high. This phenomenon could be related to the high tyrosine content in tofu and the particular microorganisms in the environment. As one of the major flavor components in the fermentation of stinky tofu, esters not only influenced its quality but also endowed it with a unique fermentation flavor (He et al., 2020). These compounds were mainly produced by the reaction of free fatty acids with ethanol (Yao et al., 2021). The esters generated from the short-chain acids emitted a fruity aroma, while those from the long-chain acids gave off a fat smell (Chen et al., 2022). The principal esters of Jianshui stinky tofu were ethyl oleate and ethyl linoleate. Most of the ethyl esters gave off fruity, woody, and sweet aromas, which softened the overall aroma of stinky tofu and substantially impacted its flavor development (Li et al., 2023).

### 3.6 Metabolic mechanisms for the formation of characteristic volatile flavor compounds

During solid-state fermentation, characteristic volatile flavor compounds accumulated dynamically. This process was driven by the combined action of precursor substrate catabolism and the secondary metabolism of dominant microorganisms. Nitrogen-containing and aromatic volatile compounds, including indole and various phenols, were mainly produced through microbial catabolism of amino acids (Huang et al., 2025). Indole was primarily synthesized via microbial catabolism of L-tryptophan, a reaction mediated by tryptophanase. This enzyme catalyzed a β-elimination reaction of L-tryptophan to produce indole, pyruvate and ammonia (Lee and Lee, 2010). Phenols came largely from the catabolism of the aromatic amino acids phenylalanine and tyrosine. Microbial enzymes first deaminated and decarboxylated these amino acids to generate phenolic acid intermediates. Those intermediates then underwent further decarboxylation, oxidation and reduction reactions to form volatile phenols such as methylphenol and ethylphenol (Yang et al., 2025). Oxidative degradation of lipids was the core metabolic pathway for the formation of aliphatic flavor compounds, including methyl ketones, unsaturated alcohols, furans, and fatty acid ethyl esters. In this study, we observed high abundance of methyl ketones (2-octanone, 3-octanone) and the unsaturated alcohol (1-octen-3-ol). These compounds originated mainly from linoleic and linolenic acids present in the tofu matrix, and were produced through the lipoxygenase (LOX) pathway mediated by functional microorganisms (Xiao et al., 2024). 2-pentylfuran, a characteristic flavor compound of stinky tofu, was mainly formed via two synergistic biochemical pathways: lipid oxidation and Maillard reaction. The primary pathway was the oxidative degradation of linoleic acid. Lipoxygenase oxidizes linoleic acid to form hydroperoxide intermediates, which then underwent cyclization and dehydration to directly generate 2-pentylfuran. The secondary pathway was the Maillard reaction. Aldehyde intermediates from lipid oxidation react with amino compounds from amino acid catabolism in this reaction (Du et al., 2022; Zheng et al., 2022). Through condensation and cyclization, furan heterocyclic compounds are formed. The synergistic effect of these two pathways drove the continuous accumulation of 2-pentylfuran during fermentation. Ethyl esters such as ethyl oleate and ethyl linoleate imparted an elegant fruity and floral aroma to the final product. Their core formation mechanism involved the esterification reaction catalyzed by microbial esterases (Li et al., 2023). Ethanol was produced through microbial carbohydrate metabolism, while free fatty acids (oleic acid and linoleic acid) were released from lipid degradation. Under the catalysis of extracellular esterases secreted by microorganisms, ethanol and these free fatty acids underwent esterification to form fatty acid ethyl esters (Xiao et al., 2022). In this study, the formation of the characteristic flavor profile was driven by the synergistic action of three core microbial-mediated metabolic pathways: amino acid catabolism, lipid oxidative degradation, and carbohydrate metabolism.

### 3.7 Analysis of the composition of flavor

**Figure 4:**
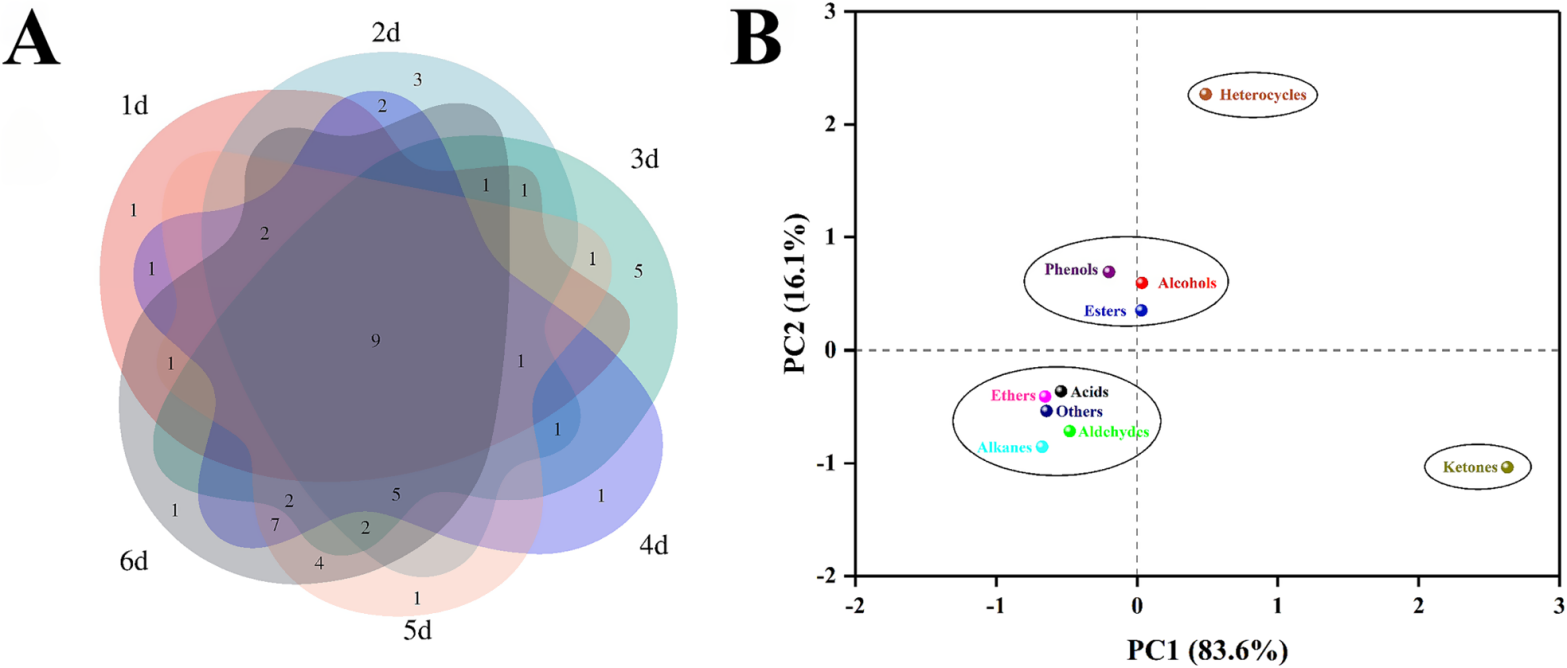
Multivariate statistical analysis of volatile flavor substances in stinky tofu. Venn diagram of volatile substances during fermentation **(A)** and score scatter plot for the principal component analysis model **(B)**.

Venn diagrams were drawn based on the number of volatile substance types during the fermentation of stinky tofu (Figure 4A). Only nine compounds were present throughout the fermentation cycle, and 43 flavor substances appeared on the fifth and sixth days. This suggested that the extension of fermentation time greatly enriched the variety of flavor substances in stinky tofu. The principal component analysis (PCA) of volatiles at different fermentation times explained 99.7% of the total variation. The first principal component (PC1) accounted for 83.6% of the total variation, with larger positive values associated with different fermentation times. The second principal component (PC2) accounted for 16.1% of the total variation. The four circles were plotted on the PCA scatter plot (Figure 4B). Ketones had the highest content in PC1, indicating that their content was higher in all samples. Heterocycles had the highest content in PC2, indicating that their content was higher in the later stages of fermentation and lower in the early stages. The contents of phenols, alcohols, and esters increased somewhat with the fermentation time. Acids, ethers, aldehydes, and alkanes were kept at low concentrations throughout fermentation.

### 3.8 Microbial analysis during fermentation process

**Figure 5:**
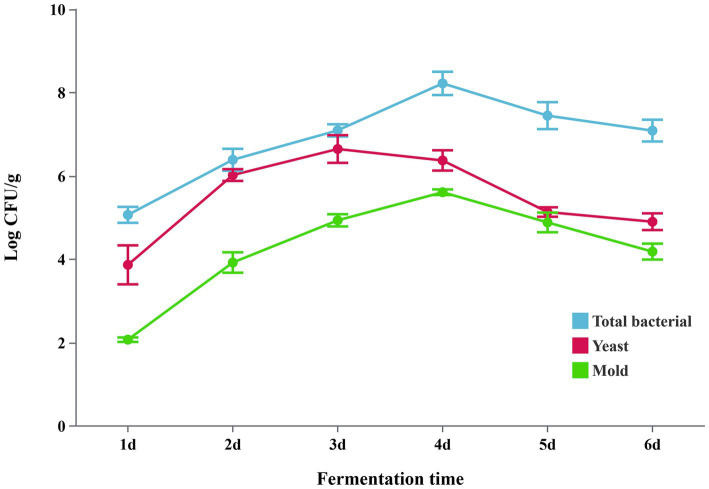
Microbial counts of total bacterial and yeast and molds (F) during the fermentation of stinky tofu.

During the initial fermentation stage, microbial populations increase rapidly. As the fermentation time extends, the microbial count in Jianshui stinky tofu tends to stabilize. In the later fermentation phase, due to the competitive relationship among microbial species and the secretion of related metabolic products, the number of microorganisms shows a downward trend (Figure 5) (Machado et al., 2021). Protease-producing bacteria hydrolyze casein into tyrosine, forming distinct proteolytic zones around their colonies (Chen et al., 2025). Using the zone-of-clear method for preliminary screening of protease-producing dominant strains, it was found that eight bacterial strains formed distinct transparent circles. Among them, strain M1-3 demonstrated the highest enzyme activity, featuring a zone-to-colony diameter ratio of 2.46 (Table 2).

**Table 2:** Preliminary screening results of protease production capacity of dominant bacteria.

Isolates	Clear zone diameter (mm)	Colony diameter (mm)	Clear zone diameter/colony diameter
B1-1	7.12	4.36	1.63
B2-3	7.56	5.16	1.47
Y1-2	12.21	8.51	1.43
Y1-4	11.51	8.64	1.33
Y1-5	9.15	6.52	1.40
M1-1	22.53	21.41	1.05
M1-2	17.43	15.47	1.13
M1-3	38.41	15.61	2.46

**Figure 6:**
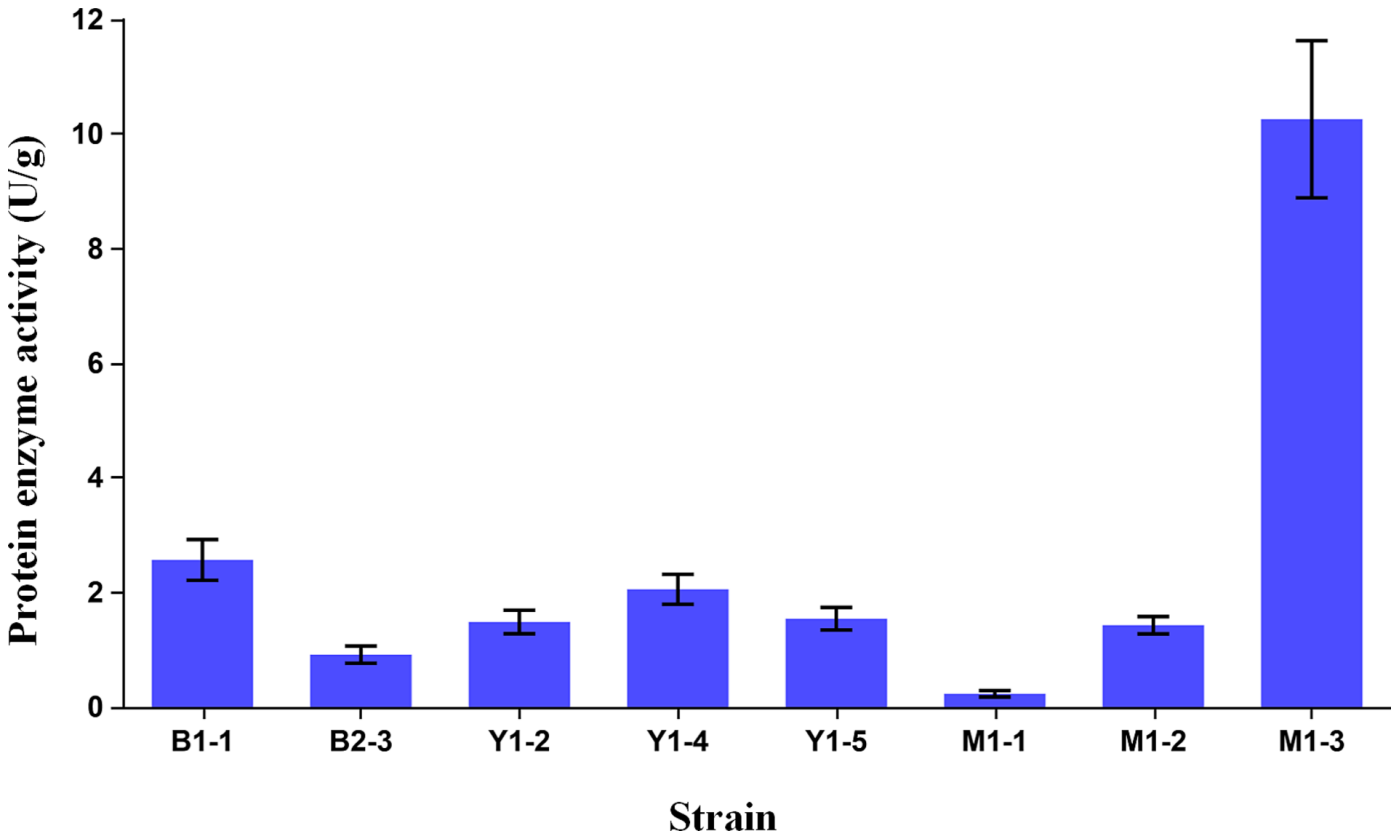
Protease activity of the rescreening strains.

**Figure 7:**
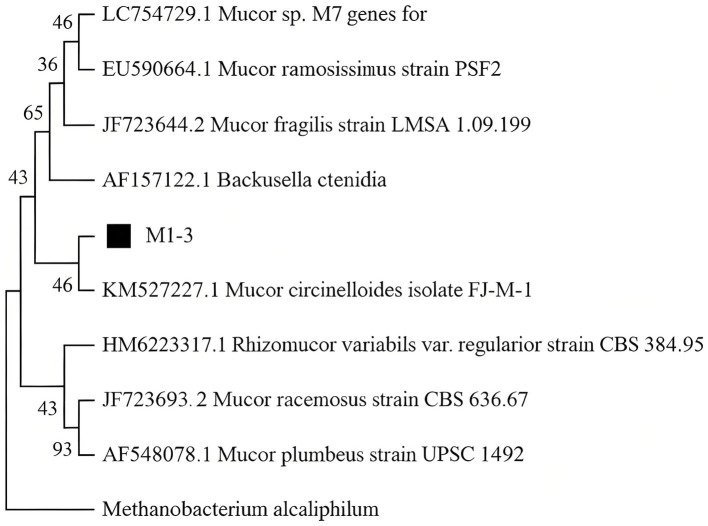
Phylogenetic tree of strains M1-3.

Protease activity was measured for these eight strains, confirming strain M1-3 as the most active at 10.26 U/g, consistent with the preliminary screening results (Figure 6). Sequence results underwent BLAST homology comparison against the NCBI database. The ITS sequence of a strain with 100% homology was downloaded and jointly constructed with the sequenced strain’s phylogenetic tree using MEGA7 software, as shown in Figure 6. M1-3 exhibited the highest homology within the same branch as *Mucor circinelloides* (Figure 7). Combined with morphological characteristics, M1-3 was identified as *Mucor circinelloides*. This fungus could produce both β-glucosidase and esterase. Through the action of these enzymes, the glycosidic bonds of phenols and flavonoids present in the tofu matrix were broken, and released free active components from bound substrates. This process significantly increased the total phenol and flavonoid contents (Lee et al., 2024). In addition, Mucor circinelloides also secreted extracellular esterases. These enzymes catalyzed the esterification reaction between free fatty acids and ethanol (Gong et al., 2025). The reaction generates ethyl esters, which were core flavor substances of stinky tofu.

## 4 Conclusion

This study systematically characterized the dynamic changes in basic physicochemical properties, nutrient components, and volatile flavor profiles during the fermentation of Jianshui stinky tofu, and isolated the dominant functional strain from this fermentation system. The main quantitative findings are summarized below. Fermentation significantly enhanced the nutritional quality of stinky tofu. By the end of the process, the total free amino acid content increased by 4-fold, with a notable increase in essential amino acid content as well. The total phenolics and total flavonoids increased by 29.84 and 162.02%, respectively. Across the whole fermentation, we identified 53 volatile flavor compounds. Ketones and heterocyclics stood out as the core flavor components. We also confirmed that the high abundance of phenols played a major role in the characteristic flavor of Jianshui stinky tofu. The dominant strain *Mucor circinelloides* with high protease-producing activity was isolated and identified. This organism appears to be the key functional microorganism driving substrate hydrolysis and product quality formation during fermentation. These results provide fundamental data for clarifying the quality formation mechanism of traditional Jianshui stinky tofu. Moving forward, we plan to apply multi-omics approaches to elucidate the microbe-metabolite interaction networks in the fermentation system. We will also test the performance of this of the functional starter culture under industrial-scale production conditions, to support the standardized and large-scale production of Jianshui stinky tofu.

## Data Availability

The raw data supporting the conclusions of this article will be made available by the authors without undue reservation. The Mucor circinelloides M1-3 ITS rRNA gene sequence is available at the NCBI GenBank with accession number: PZ447490.

## References

[ref1] Cao Z.-H. Green-Johnson J. M. Buckley N. D. Lin Q.-Y. (2019). Bioactivity of soy-based fermented foods: a review. Biotechnol. Adv. 37, 223–238. doi: 10.1016/j.biotechadv.2018.12.001, 30521852

[ref2] Chen C. Li J. Ahmed Z. Deng C. Wang F. Hu J. . (2025). Dynamic interplay between core functional microbial succession and flavor metabolite formation in naturally fermented sufu: a multi-omics perspective. Food Biosci. 71:107094. doi: 10.1016/j.fbio.2025.107094

[ref3] Chen Z. Song J. Ren L. Wang H. Zhang Y. Suo H. (2023). Effect of the succession of the microbial community on physicochemical properties and flavor compounds of Mucor wutungkiao-fermented sufu. Food Biosci. 51:102345. doi: 10.1016/j.fbio.2022.102345

[ref4] Chen Y. Wang Y. Chen J. Tang H. Wang C. Li Z. . (2020). Bioprocessing of soybeans (*Glycine max L.*) by solid-state fermentation with *Eurotium cristatum* YL-1 improves total phenolic content, isoflavone aglycones, and antioxidant activity. RSC Adv. 10, 16928–16941. doi: 10.1039/C9RA10344A, 35496929 PMC9053166

[ref5] Chen Z. Zhang C. Du H. Chen C. Xue Q. Hu Y. (2022). Effect of starter cultures on dynamics succession of microbial communities, physicochemical parameters, enzyme activities, tastes and volatile flavor compounds during sufu fermentation. Food Chem. Adv. 1:100057. doi: 10.1016/j.focha.2022.100057

[ref6] Cui Y. Lu H. Li Z. Tian Z. Yu M. Deng D. . (2022). Effects of citrus extract on number of microbes and metabolites in cecum of piglets. Chin. J. Anim. Nutr. 34, 2283–2291. doi: 10.3969/j.issn.1006-267x.2022.04.024

[ref7] Di Gioia D. Strahsburger E. de Lopez Lacey A. M. Bregola V. Marotti I. Aloisio I. . (2014). Flavonoid bioconversion in *Bifidobacterium pseudocatenulatum* B7003: a potential probiotic strain for functional food development. J. Funct. Foods 7, 671–679. doi: 10.1016/j.jff.2013.12.018

[ref8] Diez-Simon C. Eichelsheim C. Mumm R. Hall R. D. (2020). Chemical and sensory characteristics of soy sauce: a review. J. Agric. Food Chem. 68, 11612–11630. doi: 10.1021/acs.jafc.0c04274, 32880168 PMC7581291

[ref9] Du L. Ro K.-S. Zhang Y. Tang Y.-J. Li W. Xie J. . (2022). Effects of *Lactiplantibacillus plantarum* X7021 on physicochemical properties, purines, isoflavones and volatile compounds of fermented soymilk. Process Biochem. 113, 150–157. doi: 10.1016/j.procbio.2021.12.028

[ref10] Dulf F. V. Vodnar D. C. Dulf E.-H. Toşa M. I. (2015). Total phenolic contents, antioxidant activities, and lipid fractions from berry pomaces obtained by solid-state fermentation of two Sambucus species with *Aspergillus niger*. J. Agric. Food Chem. 63, 3489–3500. doi: 10.1021/acs.jafc.5b00520 25787023

[ref11] Dulf F. V. Vodnar D. C. Socaciu C. (2016). Effects of solid-state fermentation with two filamentous fungi on the total phenolic contents, flavonoids, antioxidant activities and lipid fractions of plum fruit (*Prunus domestica* L.) by-products. Food Chem. 209, 27–36. doi: 10.1016/j.foodchem.2016.04.016, 27173530

[ref12] Fan Z. Qing Q. Yin J. He C. Hu Y. Chen Y. . (2025). Metabolites of epigallocatechin gallate and changes in antioxidant activity through biotransformation with *Eurotium cristatum* during liquid-state fermentation. Food Chem. X 29:102618. doi: 10.1016/j.fochx.2025.102618, 40583894 PMC12205767

[ref13] Gao X. Liu E. Zhang J. Yang M. Chen S. (2019). Effects of sonication during moromi fermentation on antioxidant activities of compounds in raw soy sauce. LWT 116:108605. doi: 10.1016/j.lwt.2019.108605

[ref14] Gao X. Liu E. Zhang J. Yang L. Huang Q. Chen S. . (2020). Accelerating aroma formation of raw soy sauce using low intensity sonication. Food Chem. 329:127118. doi: 10.1016/j.foodchem.2020.127118, 32512391

[ref15] Gong R. Zalán Z. Song J. Suo H. (2025). Microbial fermentation of soybeans: synergistic enhancement of bioactivity and sensory properties. Food Chem. X 30:102924. doi: 10.1016/j.fochx.2025.102924, 41049771 PMC12491728

[ref16] Guo W. Chen M. Cui S. Tang X. Zhang Q. Zhao J. . (2023). Dynamics changes in physicochemical properties, volatile metabolites, non-volatile metabolites, and physiological functions of barley juice during *Bifidobacterium infantis* fermentation. Food Chem. 407:135201. doi: 10.1016/j.foodchem.2022.135201, 36525807

[ref17] He R.-Q. Wan P. Liu J. Chen D.-W. (2020). Characterisation of aroma-active compounds in Guilin Huaqiao white sufu and their influence on umami aftertaste and palatability of umami solution. Food Chem. 321:126739. doi: 10.1016/j.foodchem.2020.126739, 32259730

[ref18] He J. Xie J. Liu J. Cheng M. Jiang L. Xu R. (2016). Changes trend of volatile components in stinky tofu brine during the fermentation. China Brewing 35:79. doi: 10.11882/j.issn.0254-5071.2016.07.017

[ref19] Huang Y. Peng X. Chen Y. Wang Y. Ma J. Zhu M. . (2025). Decoding the dynamic evolution of volatile organic compounds of dark tea during solid-state fermentation with *Debaryomyces hansenii* using HS-SPME-GC/MS, E-nose and transcriptomic analysis. LWT 223:117765. doi: 10.1016/j.lwt.2025.117765

[ref20] Ji X. (2020). Investigation of the volatile components in commercial sufu (Chinese fermented soybean curd) based on HS-SPME/GC-MS combined with multivariate statistical analysis. J. Food Process. Preserv. 44:e14309. doi: 10.1111/jfpp.14309

[ref21] Jia X. Zhou Q. Wang J. Liu C. Huang F. Huang Y. (2019). Identification of key aroma-active compounds in sesame oil from microwaved seeds using E-nose and HS-SPME-GC×GC-TOF/MS. J. Food Biochem. 43:e12786. doi: 10.1111/jfbc.12786, 31608473

[ref22] Lee H. Y. Kim H. S. Kim M. J. Seo Y. H. Cho D. Y. Lee J. H. . (2024). Comparison of primary and secondary metabolites and antioxidant activities by solid-state fermentation of *Apios americana* Medikus with different fungi. Food Chem. 461:140808. doi: 10.1016/j.foodchem.2024.140808, 39151342

[ref23] Lee J.-H. Lee J. (2010). Indole as an intercellular signal in microbial communities. FEMS Microbiol. Rev. 34, 426–444. doi: 10.1111/j.1574-6976.2009.00204.x 20070374

[ref24] Li X. Gao J. Simal-Gandara J. Wang X. (2021). Effect of fermentation by *Lactobacillus acidophilus* CH-2 on the enzymatic browning of pear juice. LWT 147:111489. doi: 10.1016/j.lwt.2021.111489

[ref25] Li Y. Yuan L. Liu H. Liu H. Zhou Y. Li M. . (2023). Analysis of the changes of volatile flavor compounds in a traditional Chinese shrimp paste during fermentation based on electronic nose, SPME-GC-MS and HS-GC-IMS. Food Sci. Human Wellness 12, 173–182. doi: 10.1016/j.fshw.2022.07.035

[ref26] Liu X. Qian M. Shen Y. Qin X. Huang H. Yang H. (2021). An high-throughput sequencing approach to the preliminary analysis of bacterial communities associated with changes in amino acid nitrogen, organic acid and reducing sugar contents during soy sauce fermentation. Food Chem. 349:129131. doi: 10.1016/j.foodchem.2021.129131 33581434

[ref27] Liu Y. Song H. Luo H. (2018). Correlation between the key aroma compounds and gDNA copies of *Bacillus* during fermentation and maturation of natto. Food Res. Int. 112, 175–183. doi: 10.1016/j.foodres.2018.06.033 30131126

[ref28] Luo T. Xie Y. Dong Y. Liu A. Dong Y. (2017). Quality assessment of soy sauce using underivatized amino acids by capillary electrophoresis. Int. J. Food Prop. 20, S3052–S3061. doi: 10.1080/10942912.2017.1402028

[ref29] Machado D. Maistrenko O. M. Andrejev S. Kim Y. Bork P. Patil K. R. . (2021). Polarization of microbial communities between competitive and cooperative metabolism. Nat. Ecol. Evol. 5, 195–203. doi: 10.1038/s41559-020-01353-4, 33398106 PMC7610595

[ref30] Prado F. G. Pagnoncelli M. G. B. de Melo Pereira G. V. Karp S. G. Soccol C. R. (2022). Fermented soy products and their potential health benefits: a review. Microorganisms 10:1606. doi: 10.3390/microorganisms10081606, 36014024 PMC9416513

[ref31] Qiao Y. Zhang K. Zhang Z. Zhang C. Sun Y. Feng Z. (2022). Fermented soybean foods: a review of their functional components, mechanism of action and factors influencing their health benefits. Food Res. Int. 158:111575. doi: 10.1016/j.foodres.2022.111575, 35840260

[ref32] Rizzo G. (2020). The antioxidant role of soy and soy foods in human health. Antioxidants 9:635. doi: 10.3390/antiox9070635, 32708394 PMC7402135

[ref33] Samtiya M. Aluko R. E. Dhewa T. (2020). Plant food anti-nutritional factors and their reduction strategies: an overview. Food Prod. Process. Nutr. 2:6. doi: 10.1186/s43014-020-0020-5

[ref34] Shin D. Jeong D. (2015). Korean traditional fermented soybean products: Jang. J. Ethnic Foods 2, 2–7. doi: 10.1016/j.jef.2015.02.002

[ref35] Suleman R. Hui T. Wang Z. Liu H. Zhang D. (2020). Comparative analysis of charcoal grilling, infrared grilling and superheated steam roasting on the colour, textural quality and heterocyclic aromatic amines of lamb patties. Int. J. Food Sci. Technol. 55, 1057–1068. doi: 10.1111/ijfs.14388

[ref36] Sun C. Yu Q. Song C. Zhao R. Chen F. (2013). Study on the changes of free amino acids in spiced beef during processing. Food Res Dev:9. doi: 10.3969/j.issn.1005-6521.2013.24.003

[ref37] Tamang J. P. (2015a). “Health benefits of Tempe,” in Health Benefits of Fermented Foods and Beverages, (Boca Raton, FL: CRC Press), 386–409.

[ref38] Tamang J. P. (2015b). Naturally fermented ethnic soybean foods of India. J. Ethnic Foods 2, 8–17. doi: 10.1016/j.jef.2015.02.003

[ref39] Tian Z. Ameer K. Shi Y. Yi J. Zhu J. Kang Q. . (2022). Characterization of physicochemical properties, microbial diversity and volatile compounds of traditional fermented soybean paste in Henan province of China. Food Biosci. 50:102045. doi: 10.1016/j.fbio.2022.102045

[ref40] Vagadia B. H. Vanga S. K. Raghavan V. (2017). Inactivation methods of soybean trypsin inhibitor – a review. Trends Food Sci. Technol. 64, 115–125. doi: 10.1016/j.tifs.2017.02.003

[ref41] Vermeulen A. Daelman J. Steenkiste J. V. Devlieghere F. (2012). Screening of different stress factors and development of growth/no growth models for *Zygosaccharomyces rouxii* in modified Sabouraud medium, mimicking intermediate moisture foods (IMF). Food Microbiol. 32, 389–396. doi: 10.1016/j.fm.2012.07.019, 22986205

[ref42] Wang S. Feng W. Quan Y. Li Q. Dauphin G. Huang W. . (2022). A heterogeneous double ensemble algorithm for soybean planting area extraction in Google earth engine. Comput. Electron. Agric. 197:106955. doi: 10.1016/j.compag.2022.106955

[ref43] Wang L. Luo Y. Wu Y. Liu Y. Wu Z. (2018). Fermentation and complex enzyme hydrolysis for improving the total soluble phenolic contents, flavonoid aglycones contents and bio-activities of guava leaves tea. Food Chem. 264, 189–198. doi: 10.1016/j.foodchem.2018.05.035, 29853365

[ref44] Wu J. Tian T. Liu Y. Shi Y. Tao D. Wu R. . (2018). The dynamic changes of chemical components and microbiota during the natural fermentation process in Da-Jiang, a Chinese popular traditional fermented condiment. Food Res. Int. 112, 457–467. doi: 10.1016/j.foodres.2018.06.021, 30131157

[ref45] Wu S. Yan Q. Luo K. Liu H. Yang S. Jiang Z. . (2020). Optimization of fermentation conditions for *Bacillus Amyloliquefaciens* fermented tofu production and research on nutritional and functional properties. J. Chinese Cereals Oils Assoc. 35, 134–140. doi: 10.3969/j.issn.1003-0174.2020.02.023

[ref46] Xiao Y. Chen H. Wang Y. Ma J. Hou A. Wang Y. . (2024). Characterization and discrimination of volatile organic compounds and α-glucosidase inhibitory activity of soybeans (*Glycine max* L.) during solid-state fermentation with *Eurotium cristatum* YL-1. Curr. Res. Food Sci. 9:100854. doi: 10.1016/j.crfs.2024.100854, 39386052 PMC11462225

[ref47] Xiao Y. Huang Y. Chen Y. Zhu M. He C. Li Z. . (2022). Characteristic fingerprints and change of volatile organic compounds of dark teas during solid-state fermentation with *Eurotium cristatum* by using HS-GC-IMS, HS-SPME-GC-MS, E-nose and sensory evaluation. LWT 169:113925. doi: 10.1016/j.lwt.2022.113925

[ref48] Xiao Y. Wang L. Rui X. Li W. Chen X. Jiang M. . (2015). Enhancement of the antioxidant capacity of soy whey by fermentation with *Lactobacillus plantarum* B1–6. J. Funct. Foods 12, 33–44. doi: 10.1016/j.jff.2014.10.033

[ref49] Xu L. Du B. Xu B. (2015). A systematic, comparative study on the beneficial health components and antioxidant activities of commercially fermented soy products marketed in China. Food Chem. 174, 202–213. doi: 10.1016/j.foodchem.2014.11.014 25529671

[ref50] Yamamoto N. Shoji M. Hoshigami H. Watanabe K. Takatsuzu T. Yasuda S. . (2019). Antioxidant capacity of soymilk yogurt and exopolysaccharides produced by lactic acid bacteria. Biosci. Microbiota Food Health 38, 97–104. doi: 10.12938/bmfh.18-017, 31384521 PMC6663512

[ref51] Yang C. Lu X. Ho C.-T. Ma J. Wang Y. Chen Y. . (2025). Comparison of solid-state fermentation with different *Bacillus* species on the volatile organic compounds and non-volatile metabolites of dark teas. Food Res. Int. 214:116574. doi: 10.1016/j.foodres.2025.116574, 40467197

[ref52] Yang Y. Niu C. Shan W. Zheng F. Liu C. Wang J. . (2021a). Physicochemical, flavor and microbial dynamic changes during low-salt doubanjiang (broad bean paste) fermentation. Food Chem. 351:128454. doi: 10.1016/j.foodchem.2020.128454 33652296

[ref53] Yang Y. Wang B. Fu Y. Shi Y. Chen F. Guan H. . (2021b). HS-GC-IMS with PCA to analyze volatile flavor compounds across different production stages of fermented soybean whey tofu. Food Chem. 346:128880. doi: 10.1016/j.foodchem.2020.128880 33418415

[ref54] Yao W. Cai Y. Liu D. (2022). Analysis of flavor formation during production of Dezhou braised chicken using headspace-gas chromatography-ion mobility spec-trometry (HS-GC-IMS). Food Chem. 370:130989. doi: 10.1016/j.foodchem.2021.130989 34509944

[ref55] Yao D. Xu L. Wu M. Wang X. Zhu L. Wang C. (2021). Effects of microbial community succession on flavor compounds and physicochemical properties during CS sufu fermentation. LWT 152:112313. doi: 10.1016/j.lwt.2021.112313

[ref56] Yu J. Lu K. Zi J. Yang X. Zheng Z. Xie W. (2022). Halophilic bacteria as starter cultures: a new strategy to accelerate fermentation and enhance flavor of shrimp paste. Food Chem. 393:133393. doi: 10.1016/j.foodchem.2022.133393, 35688091

[ref57] Zaheer K. Humayoun Akhtar M. (2017). An updated review of dietary isoflavones: nutrition, processing, bioavailability and impacts on human health. Crit. Rev. Food Sci. Nutr. 57, 1280–1293. doi: 10.1080/10408398.2014.989958 26565435

[ref58] Zhang S. Shi Y. Zhang S. Shang W. Gao X. Wang H. (2014). Whole soybean as probiotic lactic acid bacteria carrier food in solid-state fermentation. Food Control 41, 1–6. doi: 10.1016/j.foodcont.2013.12.026

[ref59] Zhang Y. Zeng T. Wang H. Song J. Suo H. (2021). Correlation between the quality and microbial community of natural-type and artificial-type Yongchuan Douchi. LWT 140:110788. doi: 10.1016/j.lwt.2020.110788

[ref60] Zhao G. Li J. Zheng F. Yao Y. (2021). The fermentation properties and microbial diversity of soy sauce fermented by germinated soybean. J. Sci. Food Agric. 101, 2920–2929. doi: 10.1002/jsfa.10924, 33159694

[ref61] Zheng Z. Zhang M. Liu W. Liu Y. (2022). Effect of beef tallow, phospholipid and microwave combined ultrasonic pretreatment on Maillard reaction of bovine bone enzymatic hydrolysate. Food Chem. 377:131902. doi: 10.1016/j.foodchem.2021.131902, 34974407

[ref62] Zhu W. Luan H. Bu Y. Li X. (2019). Flavor characteristics of shrimp sauces with different fermentation and storage time. LWT 110, 142–151. doi: 10.1016/j.lwt.2019.04.091

